# Gout in the “Anonymus Parisinus”

**DOI:** 10.1007/s40620-025-02444-w

**Published:** 2025-10-30

**Authors:** Natale Gaspare De Santo, Luca S. De Santo, Carmela Bisaccia

**Affiliations:** 1 University of Campania Luigi Vanvitelli, Naples, Italy; 2 Translational Medicine Department, Luigi Vanvitelli University Hospital, AO dei Colli Monaldi. Adult Cardiac Surgery, Naples, Italy; 3 Naples, Italy

**Keywords:** Gout, Anonymus Parisinus, Ivan Garofalo, Corpus Hippocraticum, Galen, Renaissance

## Abstract

**Background:**

Gout, a disease already described in the** “**Anonymus Parisinus Darembergii sive Fuchsii”, one of the two surviving Greek medical manuscripts of the first century CE, is reviewed in an effort to trace the timeline of the knowledge of the disease between the Corpus Hippocraticum and the Renaissance.

**Methods:**

The treatise exists in four manuscripts of varying lengths: two are located in Paris, one in Vienna, and one in London. The study was conducted using the 1997 Leiden critical edition by Ivan Garofalo, which unifies all four manuscripts (“Anonymi Medici. *De Morbis acutis et chronicis*”), and was translated into English by Brian Fuchs. The treatise consists of 51 sections (*a capite ad calcem, *head to heel), in which the description of sixteen acute diseases precede that of thirty-five chronic diseases. The chapter on diseases affecting the joints precedes the last chapter, that describes elephantiasis. The text on gout consists of 945 words covering causes (46 words), signs (138 words) and therapy.

**Results:**

The causes of gout are attributed to bilious humors and phlegm, as described by the "Ancients”. The signs include inflammation and severe pain, typically beginning in the great toe (later known as *podagra*), but can extend to affect the entire leg, hands (referred to as *cheiragra*), or other joints, indicating a broader condition of arthritis. Pain is more tolerable when swelling coexists. Therapy is based on immediate bloodletting, dietary restrictions, and abstention from meat, wine and venery.

**Conclusions:**

Gout in the “Anonymus Parisinus” allows a full understanding of gout in the centuries between the Corpus Hippocraticum and Galen.

**Graphical Abstract:**

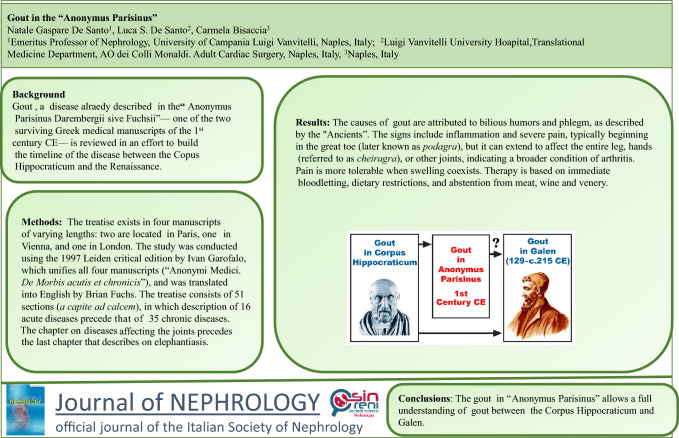

## Introduction

Gout is the oldest recorded form of chronic inflammatory arthritis affecting humankind. Its roots stretch back to 2640 BCE. It has been known as podagra (foot-grabber) from the Greek stem (pous, meaning foot and agra, meaning seizure). The term gout was introduced by Randolphus of Bocking (1197–1258), who pointed to the Latin stem *gutta* (drop).

Gout is a systemic disease caused by the deposition of sodium monourate crystals in peripheral joints and periarticular tissues caused by hyperuricemia at or above 6.8 mg/dl. Hyperuricemia is due to renal overload, renal underexcretion, or a combination of both [1–4]. Urate deposition causes the release of cytokine IL-1β [4], the key mediator of the acute attack. At onset, gout typically affects one joint, often the first metatarsophalangeal joint of the great toe, and is usually self-limited, healing within two weeks. Flare-ups affect two or more joints, becoming a chronic disease with tophi and erosion. Diagnosis is based on symptoms, signs, polarization microscopy, or fine needle aspiration of tophi [5, 6].

An effective cure was made available in the fifth century CE by Severus Iatrosophista, Theodosius the Philosopher, and Jacobus Psychrestos [7]. They introduced the use of hermodactyl, a root rich in colchicine, previously used as a cathartic. It was the main drug for the treatment of gout until the advent of allopurinol in 1966.

Gout is a disease of distinction [8, 9], historically segregating individuals by culture and class, but it is now spreading to lower socioeconomic groups [10]. Consequently, gout can be viewed as the first non-communicable disease that can be explained through the lens of Omran’s theory of epidemiological transition [11, 12]. Lifestyle, income, and education are key elements. Educated people, when made aware of the risks, are more prone to change their lifestyles, whereas poor and uneducated people may not [13]. Those who do not understand the risk factors are more likely to experience higher morbidity and mortality, while those with access to education are more likely to avoid the consequences [14].

## The goal of this study

We have embarked on a project aiming to define the timeline of gout from the *Corpus Hippocraticum* to the Renaissance, including a total of twenty-five authors, one of whom is unknown (Table 1). We have already analyzed [14–17] the contributions of Galen (129-circa 216 CE) [14], Alexander of Tralles (525–605 CE) [15], Hippocrates (460–370 BCE), and Galen (129–216/7 CE), and have translated into English the treatise on gout by Rufus of Ephesus (80–150 CE).

**Table 1:** List of authors, 25 known and 1 unknown (Anonymus Parisinus Darembergii sive Fuchsii), studied for a project on the timeline of gout from Corpus Hippocraticum to the Renaissance

Authors	Lifespan
Hippocrates	460–370 BCE
Theophrastus	371–287 BCE
Nicander	1^3^rd-2nd c. BCE
Celsus	25 BCE-50 CE
Aretaeus of Cappadocia	1st half 1st c. CE
Scribonius Largus	1st c. CE
Dioscorides of Anazarbus	1st c. CE
Anonymus Parisinus Darembergii sive Fuchsii	1st c. CE
Lucian of Samosata	c120-after 180 CE
Galen	129-c216 CE
Oribasius	c320-400/403 CE
Severus Jatrosophista	5th c. CE
Theodosius the Philosopher	5th c. CE
Jacobus Psychrestus	5th c. CE
Aëtius	mid-5th-mid-6th c. CE
Caelius Aurelianus	6th c. CE
Evagrius Scholasticus	6th c. CE
Paulus of Aegina	625–690 CE
Rhazes	865–925 CE
Avicenna	980–1037 CE
Michael Psellus	1018–1078 CE
Constatinus Africanus	c1020-before 1087
Matthaeus Platearius	died c1161
Demetrios Pepagomenos	13th c. CE
Nikolaus Myrepsos	fl*.* 1240–1280? CE
John Actuarius	end of the 14th c

*c.* century, *fl.* floruit

The aim of this study was to establish the contributions to gout of the “Anonymus Parisinus Darembergii sive Fuchsii” [18], a treatise describing medical practices and theories from the period between the works of Hippocrates (fifth - fourth century BCE) and Galen (second Century CE). We will utilize the monumental critical edition by Ivan Garofalo, translated into English by Brian Fuchs [19]), which enables contemporary scientists to fully understand an ancient author through modern language. This accessibility addresses the universal need for easier access to historical physicians’ works, which have traditionally been comprehensible only to small groups of specialists, often due to a lack of funding to meet this demand.

## The Anonymus Parisinus Darembergii sive Fuchsii

**Fig. 1:**
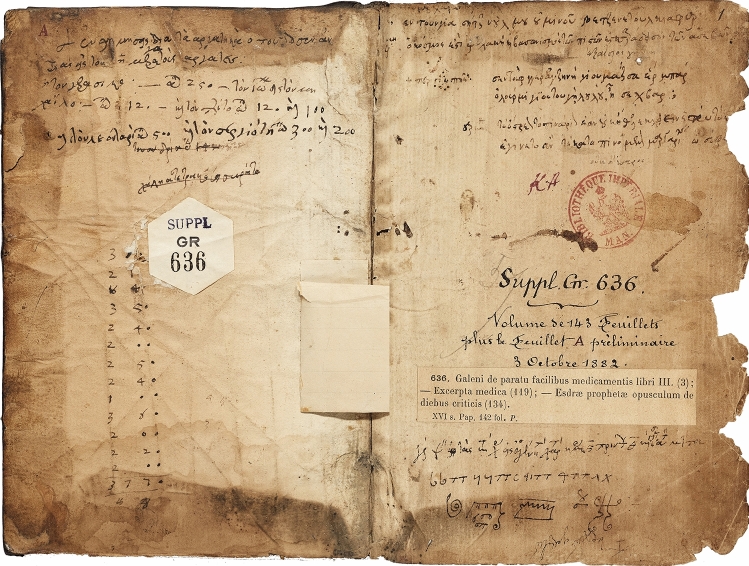
First page (recto and verso) of the *Anonymus Parisinus* supplement grec 636 of the Bibliothèque Nationale de France

A collection of four anonymous independent treatises from the first century CE outlines the signs, symptoms, and therapies of acute and chronic diseases as understood by physicians from the fifth—fourth century BCE to the second century CE, ranging from the works of Hippocrates to those of Galen. Two of these manuscripts are in Paris (*Parisinus* suppl.gr. 636 **(**Fig. 1), *Parisinus* gr. 2324), one is in London (MS 52B Wellcome collection) and the fourth is in Vienna (Vindobonensis med. gr. 37). The dating, structure (*a capite ad calcem*), and contents of the four anonymous manuscripts have been illustrated and commented on [18–23]; however, the authors remain unknown. The treatise gr.636 in the Bibliothèque Nationale in Paris is the most complete of the four. It is subdivided into fifty-one sections, starting with sixteen acute diseases followed by thirty-five chronic diseases. It is known as *Anonymus Parisinus* because it is believed to have originated from Mount Athos and to later have arrived in Paris, where it was identified and studied. It bears the title “Diagnosis concerning acute and chronic diseases” [22].

As noted by Ivan Garofalo, the “Anonymus Parisinus (AP) Darembergii sive Fuchsii” is attributed to the nameless author of the only known work, besides that of Aretaeus on acute and chronic diseases, that is preserved in Greek [19].

The title of the London manuscript is ‘A Diagnostic Discussion of the Great Physician Authors on Acute and Chronic Diseases,’ and it is subdivided into twenty-nine sections [22].

The sources of the companion treatise are Hippocrates (460–370 BCE), Diocles of Carystus (c.375-c.295 BCE), Praxagoras of Cos (fourth century BCE), and Erasistratus of Ceos (c.315-c240 BCE). They are identified in the text as “The Ancients” (as in the chapter on gout, or “ The four”. Regarding the section on elephantiasis, the source is Democritus. No source is provided for one acute disease (satyriasis) or for twelve chronic diseases [22].

The text was discovered by Mynoides Mynas in a manuscript found on Mount Athos and later transported to Paris. The first reference to it can be found in Mynas’ preface to his 1844 edition of Galen’s *‘Institutio Logica’* [20].

The "Anonymi Medici. *De morbis acutis et chronicis*,” edited with commentary by Ivan Garofalo and translated by Brian Fuchs [19], utilizes all four treatises. The result is a comprehensive work that presents acute and chronic diseases in a systematic manner, from head to heel, covering conditions from phrenitis to satyriasis (acute diseases) and from vertigo to elephantiasis (chronic diseases) [23].

The reconstruction of the texts by Ivan Garofalo [18, 19] makes full use of passages from Oribasius, Aetius of Amida, and Paul of Aegina, excerpted from *Anonymus Parisinus*. The result is a living text that provides many non-specialists, who have not studied Greek, with the opportunity to access a medical text from antiquity, compiled for practitioners, and in a modern language. “Anonymous Parisinus*”* and the companion treatises are important because they discuss both acute and chronic diseases.

As discussed by Riddle “In Chronic Diseases (*I. praef. 3*)”, Caelius Aurelianus (ca. 5th CE) said that Themison (late I^st^ BCE) first set forth treatments for chronic diseases. This anonymous work and those by Caelius Aurelianus, Aretaeus of Cappadocia (second half of 2nd CE), and, possibly, Archigenes (f*loruit* under Trajan, 98–117 CE) are the only ones known to have organized medical information under these distinctions. Indeed, the title "On acute and chronic diseases" was not in the Greek manuscript texts [23].

However, unlike Caelius Aurelianus, “Anonymus Parisinus” includes cephalea and colic in acute diseases, and at variance with Aretaeus—but in agreement with Caelius Aurelianus —includes madness and hemoptysis in chronic diseases.

Anonymus Parisinus does not provide criteria to differentiate between acute and chronic diseases, however at the end of Section XVI dedicated to satyriasis he says “We shall make an end here of the exposition of the acute diseases and now consider the collection of the chronic diseases” as reported on page 109 of the translation that is Garofaolo’s masterpiece.

## Discussion

According to Hippocrates, gout was a painful but non-fatal condition caused by bile and phlegm. It did not affect prepubertal children, eunuchs, and premenopausal women. It typically flared up in spring and autumn and could only be effectively treated during its acute phase. Once it progressed to a chronic state—with tophi and bone erosions—treatment became impossible. During the acute phase, pain relief was achieved using opium poppy and hyoscyamus. Hippocrates attempted to reinforce the body’s natural healing process through fasting, a liquid diet, purges, clysters, and emetics, along with exercise, massages, and baths. Bleeding became necessary in the presence of excess blood. Abstention from wine and sexual activity was mandatory [16].

“In Galen’s view, gout is due to fluid overflow that infiltrates nerves and causes pain. Overflowing fluid may be blood, phlegm, or a mixture of bile, blood, and phlegm. The prevailing humor is crude, mucous, and thick, and by residing in the joint, causes tophi. The nature of infiltrating humor can be diagnosed through the color of the joint, symptoms, effects of heat and cold, effects of drugs, and information related to age, diet, quantity and quality of exercise, and attitude towards baths of the patient. Treatment required immediate bloodletting by venesection at the elbow, which could be repeated. Purges, enemas, and/or emetics were additionally needed to evacuate the humor(s). Poultices played a role in draining the humor(s) as well as for their emollient-softening properties” [14].

We shall bear in mind that according to the “Corpus Hippocraticum”, gout was due to the accumulation of yellow bile, black bile, and phlegm in the blood. The humoral theory, confirmed by Galen centuries later, survived until the seventeenth century.

The section on gout in “Anonymy Medici” consists of a text of 945 words and follows the usual format. A total of 46 words describe the cause, 138 the signs. The remaining 761 words outline the therapy structured over a total of 20 guidelines. Therein, one can appreciate even the stylistic uniqueness underscored by Ivan Garofalo in his introduction concerning the use of the second plural imperative [19]. Therapy is not personalized and ends with suggestions about the start of the restorative phase [22].

Gout, according to the “Ancients” (the 4 authors utilized by “Anonymus Parisinus” to define acute and chronic diseases), “is an inflammation of the tendons of the foot joints and arises sometimes from bilious humors, sometimes from phlegmatic ones.” It manifests with violent pain originating in the big toe or the joint, and may extend to the whole leg. It is called podagra when localized in the foot, cheiragra when localized in the hands, and arthritis when affecting other joints. Pain is less severe when the process is accompanied by swelling. Treatment is based on immediate bloodletting, fasting, abstention from sex, exercise, emptying the intestines by means of enemas, emetics, and purges. Therapy also makes use of embrocations, poultices, and lenitives based on poppy heads or roots of marsh mallow.

“**1.** Bleed the patients initially, if it is possible; otherwise, evacuate with acid clyster and cure with the light treatment, as in arthritis**. 2**. In the intervals, let them adopt a long regimen with water alone, and abstain from sex; in many cases, these measures suffice to heal**. 3**. You must make the regimen gymnastic in the event of fair weather and apply vomiting, both fasting and with radishes. **4**. A sufficient length of time is six months, after which you should come back to the previous regimen little by little. **5.** If they are not healed with the aforementioned remedies, have them take hellebore and drugs with centaury and scordium, prepared for one year, as they are excellent and digestible.”

“Anonymus Parisinus”, at variance with Hippocrates, also includes patients with chronic gout:

“**6**. Treat chronic gout at the beginning with reduced food and water drinking and excretion of the belly. If they are accustomed to it, bleed them.”

“Anonymus Parisinus” is very accurate in describing simple poultices that disclose his masterly clinical skills:

“**15**. […], a simple poultice with fenugreek, or linseed, or iris, or barley meal, or bean, or vetch, or the root of marsh mallow cooked in water with honey, either plain or with linseed, or the root of wild cucumber cooked with water, first douching with such water, then applying the plaster either with vine-leaves with flower of wheat meal, or with marsh lentil, now plain, now with fine meal, and now cold, now gently warmed with < ... > and tendrils of evergreen or leaves of box-thorn with flower of meal or lentil boiled plain or the core of the mallow or of olive sprouts or of lemon or of cucumber or of ripe melon or with fresh oregano or sleepy nightshade or purslane or green pomegranate leaves, pomegranate flowers boiled with diluted vinegar or wild rue, with vinegar, both plain and with meal flower, or in vinegar with celery or figs boiled with water in such a way as to reach the density of honey, or boiled twice.”

However, clinical experience tells him that simple poultice may not reach the goal, and in this eventuality, he provides additional suggestions:

“**16**. If they are not soothed, add capsules of poppy pounded with their leaves or with the leaves of quinces or sweet pomegranate-peel cooked each apart with white wine and pounded in such a way as to take the density of barley-gruel juice, then boiled together with barley meal or bitumen pounded, with wine, or bulbs with honey or egg whites or rue with honey or root of henbane with storax, or hemlock with oxymel or horehound pounded with salt or tender cheese cut in slices and used as a plaster and frequently changed, or nettle seed and a double quantity of flax seeds cooked with vinegar lees, quicklime, and nitrum with old pig fat or juice [silphium] and storax and bitter almonds or henna oil and vinegar applied as a plaster continuously, or bread with oil and water or with honey. **17**. You must soothe their pains with decoction of marsh mallow or bitumen or sulphur, and after this, cover with a simple poultice.”

It must be underlined that guidelines **15–17** include most of the remedies of plant origin used for the treatment of gout in “Anonymus Parisinus”.

Aulus Cornelius Celsus (c. 25 BCE—c. 45 CE) also prescribed bleeding as a means to achieve both immediate and lasting well-being, potentially enduring for up to a year [24]. According to Celsus, abstention from wine and venery was mandatory for an entire year. For intolerable pain, he also recommended using the rind of poppy heads (*Papaver somniferum* L.), boiled in wine and mixed with a wax-salve infused with rose oil. Alternatively, equal parts of wax and lard can be melted together, and then mixed with the wine. As soon as this application becomes warm, it should immediately be removed and replaced with another. However, if the swellings have hardened and become painful, applying a sponge frequently soaked in oil and vinegar, or cold water, can provide relief. Alternatively, a mixture of equal parts pitch, wax, and alum may also be effective. There are also several emollients suitable alike for the hands and feet. But if the pain was too intense to tolerate anything being applied, when there is no swelling, the joint should be fomented with a sponge which has been dipped in a warm decoction of poppy-head rind, or of wild cucumber root, next the joints are smeared with saffron (*Crocus sativus*, L), poppy-heads juice (*Papaver somniferum* L.) and ewe’s milk.

In case of an acute attack, if little time has elapsed, Aretaeus of Cappadocia (mid-first century CE) suggested dietary restriction, and to purge with black hellebore *(Helleborum Cyclophyllum R.Br).* Hellebore drives down bile and phlegm. He also suggested applying unscoured sheep wool, anointing with rose-oil, and bathing with a cold sea-water sponge soaked with oxycrate. He also deemed it appropriate to prepare cataplasms made of bread, pumpkin (*Lagenaria vulgaris* Ser.), cucumber (*Colocasia antiquorum* Schott.), plantain (*Plantago* L. sp.), and rose leaves. Cataplasms made of bread and sideritis (*Sideritis scordioides* L.) mitigate pain, as do decoctions of roots of comfrey (*Tussilago farfara* L.), cinquefoil (*Potentilla reptans*, L.), and leaves of horehound (Marrobium*vulgare* L*., Marrobium creticum* Miller).

Pedanius Dioscorides of Anazarbus (first century CE) deserves a special mention since *De material medica* (cited extensively by Galen) can be defined as the book of gout. There are more than 60 passages where therapy of gout is discussed. In addition, in Book IV, 42 he introduces the Five Finger Grass known as Pentatomon or Cinquefoil pentadactylon, hermodactylon (*Potentilla reptans L.*). “The decoction of its root can …alleviate …toothaches … and those suffering in the joints and from hip ailments when drunk” [25]. He probably knew the virtue of hermodactlylum, (*Colchicum autumnale* L) but attributed its virtue to another plant.

The four humors were listed as a cause of gout in the 37-chapter treatise of Rufus of Ephesus (98–138 CE). Rufus believed bloodletting was necessary, and that it had to be carried out as soon as possible. Immediacy was particularly beneficial for plethoric individuals by sectioning the vein close to the site of inflammation. Dietary restrictions, abstention from sex, exercise, and baths were part of the armamentaria.

We have attempted to identify the plants used in the treatment of gout by “Anonymus Parisinus” (Table 2), drawing upon the seminal works of John M. Riddle [26] and Lilly Y. Beck [25] on Dioscorides, as well as Nicholas Everett’s monograph on *The alphabet of Galen* [27]. These works cover scientific literature related to plants, minerals, and animals from the third century BCE to the first century CE. With the understanding that this information is intended for historical purposes rather than therapeutic use, the table may help illustrate the plant remedies for gout detailed in the *“Anonymus Parisinus”.* Identifying evergreens can be challenging due to their diversity; the category includes cypress, pine, palm, olive, boxwood, holm oak, privet, yew, hornbeam, beech, laurel, myrtle, and rosemary, among others. In general, the vegetable remedies noted in “Anonymi Medici” align well with the medical practices of the period spanning from the fifth—fourth century BCE to the second century CE. Here are a few examples: hellebore (Hippocrates); almonds, centaury, cypress, cucumber, hellebore, poppy capsules (Celsus); purslane (Pliny); barley, figs, fenugreek henbane, marsh lentil, marsh mallow, scordium (Dioscorides). Specifically, Dioscorides used (i). a warm poultice of white willow bark and leaves (*Salix alba* L.) containing salicine; (ii). a plaster made of asphalt, sodium carbonate and barley meal (*Hordeum sp.*) as well as (iii) local application of fig pulp (*Ficus carica* L.), fenugreek flower (*Trigonella foenum graecum* L.) and vinegar; (iiii). a drachma of the root juice of asphodel (*Asphodelus ramosus* L., *A. albus* L.) [26].

**Table 2:** An attempt to identify the origins of vegetable remedies for gout by Ivan Garofalo (19)

Common name	Botanical name
Almonds (bitter)	*Prunus amigdalus* Stokes var. *amara*
Barley	*Hordeum* L. Sp
Bean	*Vicia faba* L. (LSJ)
Boxthorn, leaves	*Rhamnus cathartica* L. *Buxus Sempervirens* L
Celery	*Apium graveolens* L, var.*silvestre*
Centaury	*Centhaurea centaurium* L
Cucumber	*Cucumis sativus* L
Cypress	*Cupressus sempervirens* L
Fenugreek	*Trigonella fenumgraecum* L
Figs	*Ficus carica* L
Hellebore	*Veratrum album* L., *Hellebore nige*r L
Hemlock	*Conium Maculatum* L
Henbane (root)	*Hyosciamus niger* L
Henna (oil)	*Lawsonia inernis* L
Horehound	*Marrubium vulgare* L
Iris	*Iris florentina* L., *I. germanica* L., *l. pallida* Lamk
Lemon (sprouts)	*Citrus medica* L
Linseed	*Linum usitatissimum* (LSJ)
Mallow, core	*Malva silvestris* (LSJ)
Marsh Lentil	*Ervum lens* L. (LSJ)
Marsh mallow	*Althaea officinalis* L
Nettle (seed)	*Urtica dioica L*
Olive (sprouts)	*Olea Europea* L. var. *sativa*
Oregano (fresh)	*Origanum vulgare* L
Pomegranate*	*Punicagranatum* L
Poppy (capsules)	*Papaver somniferum*L
Purslane	*Portulaca oleracea* L
Quinces (leaves)	*Cyngonia oblonga* Miller
Rue	*Ruta graveolens* L.*, and/or R. montana L*
Scordium	*Teucrium scordium* L
Sleepy nightshade	*Withania somnifera Dun*
Storax	*Styrax officinalis* L
Vetch	*Vicia sativa L*
Vine leaves	*Vitis vinifera* L
Wheat	*Triticum L*

^*^Green leaves, flowers, and peel

## Conclusions

We are aware that a critical analysis comparing “Anonymus Parisinus” Darembergii sive Fuchsii" with other authors would be useful and would guide the reader in a more fluid manner. None of the authors listed in Table 1 commented on “Anonymus Parisinus” since the manuscript, for some mysterious reason, remained hidden in the bookshelves at Mount Athos until 1840. The advent of Galen crystallized the entire field of medicine, not just the section on gout [14], until the seventeenth century, until the advent of the first treatments with colchicine, unknown to “Anonymus Parisinus”. Thus, we are the first generation, following the “Princeps edition” by Garofalo, to study it. However, since 1997, no novelties have come to light, and further studies are warranted.

Gout, like all diseases in the “Anonymus Parisinus,” is presented with remarkable succinctness, similar to the concise style of a modern UK medical manual, where each line is purposeful, covering etiology, diagnosis, and therapy efficiently. As previously outlined, the causes and signs of the disease are minimized. Therapy is described in detail. Nothing is comparable, not only to Hippocrates and Galen but also to the 37 books by Rufus of Ephesus and the lengthy chapters by Aetius of Amida and Alexander of Tralles.

As underlined by Orly Lewis it is “a handbook”, so to speak; it does not aspire to instruct in medical theory or argue for overarching ideas concerning disease or the body, or concerning drugs and other treatments”[22].

It must be underlined that the text of the “Anonymus Parisinus” is something brought about by a work of slimming to the essential, by removal, not by adding (Sossio Giametta, Frattamaggiore 1929—Bruxelles 1924), just as the sculpture of Michelangelo Buonarroti (“Sculpture is an art which by removing, not by adding…”) described by Vasari in “Le vite dei più eccellenti pittori”. Its conciseness even surpasses that of Celsus, who dedicated 1051 words to gout, discussing even the pathogenesis of tophi. However, its conciseness is surpassed by the “Poem on Podagra” by Michael Psellus (1018–1078), in 36 lines, where we learn that a humor generated by excess meat intake spreads in the body and, because of its irritating capacity, induces a disease that may present with hardness (tophi).

Therefore, we have selected a few authors between the time of Hippocrates and that of Galen, and emphasized the importance of Dioscorides, also because of the problem of colchicine, which is thoroughly discussed. These authors do indicate that there was progress in the field of gout between Hippocrates and Galen, with contributions from various other authors, that could not be incorporated into “Anonymus Parisinus”, which was written for the physician who had to make a diagnosis and needed to know what to do when treating patients with acute and chronic disease.

In conclusion, the critical edition of “Anonymus Parisinus Dambergii sive Fuchsii”, by Ivan Garofalo, translated into English by Brian Fuchs [19], provides an excellent synopsis of the causes, signs, and treatment of gout as understood during the period of time between Hippocrates and Galen, including insights into pain. To the best of our knowledge, this is the first time that this treatise has been incorporated into the timeline of gout, spanning from the Corpus Hippocraticum to the Renaissance. The English translation from Greek of Anonymi Medici enables non-specialists who do not know ancient Greek to fully engage with the treatise in a modern language, thereby fulfilling the widespread need to comprehend its message in its entirety.

## Data Availability

The vast majority of papers have an identifier (DOI), a minority have an ISBN. Papers are easily available in the section history of medicine of any academic library. Authors are available to answer any questions.
